# Editorial: Next generation micro-physiological systems for pragmatic use in regular industrial workflow

**DOI:** 10.3389/fcell.2025.1607979

**Published:** 2025-05-29

**Authors:** Ali Kermanizadeh, David M. Brown, Wolfgang Moritz

**Affiliations:** ^1^ College of Science and Engineering, University of Derby, Derby, United Kingdom; ^2^ School of Engineering and Physical Sciences, Heriot Watt University, Edinburgh, United Kingdom; ^3^ InSphero, Schlieren, Switzerland

**Keywords:** micro-physiological systems (MPS), drug discovery, safety, liver, intestines, tumour microenvironment

## Background

Although *in vivo* models are currently preferred for use in drug discovery efficacy testing at the target validation and late lead optimization stages, *in vitro* models have gained considerable popularity for candidate selection from hit to lead and within lead optimization. Over the last 15 years micro-physiological systems (MPS) have significantly advanced and show great promise for predicting drug safety as compared to 2D cell cultures and traditional *in vivo* models. Despite these advancements in MPS there is an urgent need to improve and standardise these testing models in particular due to increased number of novel therapeutic modalities such as biologics, oligonucleotides, peptides, human antibodies, gene and cell therapy which are not effective in animal models. Advanced physiologically relevant MPS are designed to a) incorporate multiple cell populations including immune cells allowing capturing of immune responses following xenobiotic testing; b) to avoid dealing with interspecies differences between preclinical animal models and humans and c) to foster reduction and replacement of animal testing. In this Research Topic, manuscripts were invited that focused on physiological and pathophysiological industrial compatible *in vitro* MPS designed for next generation testing which demonstrated technical accessibility as well as economical and ethical/moral considerations.

## Content of the Research Topic

In this Frontiers Research Topic, a total 5 of multi-national collaborative studies were accepted including research from the United Kingdom, Germany, Netherlands and United States. We believe these global contributions highlight the importance of the manufacture, standardisation and main-stream utilisation of physiologically relevant MPS in the field of drug safety and efficacy. The authors who have contributed to this Research Topic have introduced and discussed models of the intestines, the liver and tumour microenvironment. Below, we provide a Research Topic summary of the contributions based on the different models introduced.

## Intestines

Klein et al. introduced a primary duodenal cell derived intestinal organoid model. The organoids were cultured either in proliferative or differentiated conditions before being exposed to a range of small molecule therapeutics. The authors showed a clear difference in toxicological output from the drugs dependant on exposure of proliferative or differentiated organoid models. This data demonstrated the importance of the differentiation status of intestinal cells in governance of overall drug induced toxicity assessment and the subsequent conclusions made. Despite the interesting findings in this study two limitations of the MPS were highlighted: a) only acute exposure to xenobiotics was possible and b) exposure of the xenobiotics in the basolateral surface which is not ideal considering orally administered drugs will come across the apical surface of cells in *in vivo* scenarios.

Elsewhere, Hoffman et al. discuss a high throughput cell line (Caco-2) based intestinal model established on an organ on chip platform (OrganoPlate®). Following establishing of tight junctions, the test systems were exposed to a wide range of drugs. The data showed a good correlation between the toxicological data obtained from the MPS here and historic clinical observation for the same drugs. Overall, the MPS was capable of re-capturing the intestinal barriers and offered a good alternative for assessment of drug toxicity in the GIT. As two limitations of the model: a) the model only contained a single cell type and b) only acute exposure of drugs were investigated. Overall, the data presented demonstrates that proposed MPS could be a viable candidate for early safety prediction of drug-induced damage on the intestinal barrier.

## Liver

Elcombe et al. used primary human and rat spheroids (constituted of hepatocytes, Kupffer cells and sinusoidal endothelial cells) combined with AI driven image analysis to investigate phenobarbital induced hepatocyte hypertrophy. In this approach object-based segmentation was performed on histology imagesthat enabled individual intracellular compartments and organelles to be detected e.g., nuclei, cytoplasm). Once the training and segmentation had completed the Arivis Cloud software outputted the results for each cell which included the cytoplasmic areas. As a limitation to the approach the AI assisted detection appears more appropriate as a qualitative means of investigating liver damage. Looking ahead, the authors believe that the AI technology could be efficient in measuring key events in the Bradford-Hill-based mode of action scheme which could enhance early compound development pipeline testing.

Xia et al., introduce a liver acinus MPS composed of primary healthy and diseased cells. Specifically, the MPS was constructed using wild-type and variant PNPLA3 (linked to liver disease progression) hepatocytes and non-parenchymal cells (primary sinusoidal endothelial cells, differentiated THP-1 macrophages and LX-2 stellate cells). Furthermore, and importantly the authors manipulated the cell culture media components to mimic normal, early and late metabolic syndrome conditions. The MPS were then exposed to resmetirom (a drug released for management of chronic liver disease). The data showed that the model could capture the key phenotypes of various stages of liver disease. These findings coincided with variances in drug efficacy between the wild-type and the variant PNPLA3 hepatocytes (greater resmetirom efficacy in wild-type hepatocytes). As a limitation, this model is still at the early stages of validation and in all reality not ready for high throughput usage.

Overall, the data from the manuscript suggest that the MPS could prove useful in the investigation of mechanisms of action of new drug modalities.

## Tumour microenvironment

Grotz et al. produced a tumour microenvironment in a 3D spheroid composed of tumour cells (triple-negative breast cancer cell line), primary human naïve T cells and fibroblasts (skin fibroblast cell line) in a 384 well format. The authors showed that in the triple-culture containing fibroblasts the 3D structures were tightly packed with these cells acting as a chemical physical barrier preventing infiltration of lymphocytes hence reduced T cell and tumour cell interactions. Next, the authors investigated immune checkpoint inhibition in the triple cell model. The data showed a decrease of tumour cell viability in the co-culture with T cells alone following treatment with Compound G. However, in the triple culture treatment with Compound G was significantly less effective suggesting at protective effect of the fibroblasts on the tumour cells. The authors also showed that fibroblasts express more *fibroblast activation protein α* in 3D compared to 2D. Despite the impressive work the 3D MPS lacked any morphological characterization of the spheroid model with only volumetric and size measurements. The authors believe that in the future the MPS could be extended to other tumour cell lines or be modified to incorporate primary cancer associated fibroblasts.

## Concluding remarks

The highlighted manuscripts exemplify the extensive array of potential applications for *in vitro* testing systems used for of efficacy and safety, utilizing advanced MPS derived from either primary cells or stem cell-derived organotypic cells. Furthermore, they provide insight into the current status of MPS regarding their preparedness for industrial implementation. Regardless of whether stem cells or primary cells are employed, achieving standardization poses a significant challenge due to the varied genetic and epigenetic backgrounds of donors, as well as lifestyle factors that may influence the outcomes of these investigations. In addition to demonstrating their robustness, reproducibility, and predictability, the future development of MPS must incorporate validation tests tailored to specific contexts of use. This process also necessitates a clear understanding of whether the emphasis is on accurately representing the general population, vulnerable sub-populations, or individual cases. Accomplishing this objective will enhance the design of clinical studies and facilitate regulatory acceptance and endorsement for Investigational New Drug (IND) applications and drug approvals.

